# The Indirect Effect of Age Group on Switch Costs via Gray Matter Volume and Task-Related Brain Activity

**DOI:** 10.3389/fnagi.2016.00162

**Published:** 2016-07-13

**Authors:** Jason Steffener, Yunglin Gazes, Christian Habeck, Yaakov Stern

**Affiliations:** ^1^PERFORM Center, Concordia University, MontrealQC, Canada; ^2^Centre de Recherche de l’Institut de Gériatrie de Montréal, MontréalQC, Canada; ^3^Department of Psychology, Concordia University, MontrealQC, Canada; ^4^Cognitive Neuroscience Division, Department of Neurology and Taub Institute for Research on Alzheimer’s Disease and The Aging Brain, Columbia University College of Physicians and Surgeons, New YorkNY, USA

**Keywords:** gray-matter volume, task related brain activity, mediation, aging, cognitive control

## Abstract

Healthy aging simultaneously affects brain structure, brain function, and cognition. These effects are often investigated in isolation ignoring any relationships between them. It is plausible that age related declines in cognitive performance are the result of age-related structural and functional changes. This straightforward idea is tested in within a conceptual research model of cognitive aging. The current study tested whether age-related declines in task-performance were explained by age-related differences in brain structure and brain function using a task-switching paradigm in 175 participants. Sixty-three young and 112 old participants underwent MRI scanning of brain structure and brain activation. The experimental task was an executive context dual task with switch costs in response time as the behavioral measure. A serial mediation model was applied voxel-wise throughout the brain testing all pathways between age group, gray matter volume, brain activation and increased switch costs, worsening performance. There were widespread age group differences in gray matter volume and brain activation. Switch costs also significantly differed by age group. There were brain regions demonstrating significant indirect effects of age group on switch costs via the pathway through gray matter volume and brain activation. These were in the bilateral precuneus, bilateral parietal cortex, the left precentral gyrus, cerebellum, fusiform, and occipital cortices. There were also significant indirect effects via the brain activation pathway after controlling for gray matter volume. These effects were in the cerebellum, occipital cortex, left precentral gyrus, bilateral supramarginal, bilateral parietal, precuneus, middle cingulate extending to medial superior frontal gyri and the left middle frontal gyri. There were no significant effects through the gray matter volume alone pathway. These results demonstrate that a large proportion of the age group effect on switch costs can be attributed to individual differences in gray matter volume and brain activation. Therefore, age-related neural effects underlying cognitive control are a complex interaction between brain structure and function. Furthermore, the analyses demonstrate the feasibility of utilizing multiple neuroimaging modalities within a conceptual research model of cognitive aging.

## Introduction

Age has a multi-faceted effect on many aspects of our bodies and our cognitive abilities. Declines in cognitive control and executive function are thought to underlie other age-related declines in cognition, specifically fluid ability (Baltes, 1993). One approach to investigating cognitive control is with tasks requiring dual-task processing (Kray and Lindenberger, 2000). Engagement in multiple simultaneous tasks requires task switching and this switch hinders response time with respect to performance of either in isolation (Rogers and Monsell, 1995). The performance decline when engaged in two tasks is termed switch costs. Switch costs fall into two categories, local and global. Local costs are the trial-to-trial decrements in performance, while global costs refer to block effects, namely blocks of single task conditions compared to blocks of dual task trials. Understanding the neural origins of increasing switch costs and cognitive control is an important goal for understanding the aging process (Braver and Barch, 2002; Madden et al., 2010). Understanding the neural underpinnings of performance decline on task-switching experiments provides better insight for intervention programs aimed at maintaining and improving daily life through maintenance of cognitive control (Karayanidis et al., 2010).

Neuroimaging approaches provide insight into the effects of aging on the brain. Advancing age affects many neural measures including gray matter volume and task-related brain activation. Age-related neural declines are also related to executive functioning (Van Petten et al., 2004; Sakai et al., 2012). Integration of various age-related findings into a single conceptual research model provides a better understanding of the complexities of the aging process. This approach may shed further light on the neural underpinnings of age-related cognitive decline. Furthermore, incorporating multiple modalities of neural measures to better understand cognitive aging is a current topic of discussion in the literature (Grady, 2012; Steffener and Stern, 2012; Reuter-Lorenz and Park, 2014). The present work attempts to explain age-related differences in cognitive control with full brain voxel-wise measures of gray matter volume and brain activation.

The hypothesis of this study is that age related declines in cognitive performance are the result of age-related declines in MRI derived measures of brain structure which result in altered brain function. This straightforward hypothesis has had limited explicit testing. Previous work along the same line of inquiry has focused on linking together functional and structural connectivity (via white matter measures) and age-related changes in cognition (Gold et al., 2008; Madden et al., 2010). The current work focuses on gray matter volume and brain function and incorporates the measures into an established theoretical model of cognitive aging (Grady, 2012; Steffener and Stern, 2012; Reuter-Lorenz and Park, 2014).

The hypothesized model of this study tests whether age-related differences in switch costs are partially explained by age-related declines in gray matter volume resulting in altered brain activation resulting in greater switch-costs. The model is tested with serial mediation analyses and links together three consistent observations in the aging literature: gray matter volume decline, task-related brain activity differences, and increased switch costs. This nature of this model incorporates the main hypothesis of this work along with three other possible effects. Age may indirectly affect switch costs via gray matter volume or brain activation, while holding the other measure constant. Age may also directly affect switch costs after accounting for both brain measures. Summing the three indirect effects and the direct effects is the total effect of age on switch costs. The total effect is simply the effect size of age on switch costs in the absence of any neural measures. The statistical tests in this study imply causal relationships between variables as specified in the *a priori* theoretical model. Since the data used is cross-sectional, caution is warranted when making causal claims or interpretations. The mediation analyses test whether the data fit the conceptual research model that age effects on brain volume, alter brain activity resulting in altered switch costs.

The task employed was the executive context factor (ECF) task first discussed by Koechlin et al. (2003). This initial work with young adults demonstrated that manipulations of cognitive control included activation within increasingly rostral regions of the inferior frontal cortex. Regional age-related differences in brain activation using this task, and multivariate statistics, included middle frontal gyrus, the precentral gyrus, the anterior cingulate, and the superior parietal lobule and were shown to be related to switch costs (Gazes et al., 2012) and replicated with an independent sample (Gazes et al., 2015). For further background on switch costs we refer the reader to these referenced works.

The current study tested for indirect and direct effects of age group on global switch costs with the ECF task. Based on our previous multivariate analyses with this task and the literature (DiGirolamo et al., 2001; Madden et al., 2010), we expected the current univariate analyses to demonstrate age related differences in task related brain activation within the middle prefrontal, precentral, cingulate and parietal regions. There is an expected age-related decrease in gray matter volume throughout the brain and switch costs are expected to increase with advancing age. This study integrates full brain data into a comprehensive model of cognitive aging.

## Materials and Methods

### Study Participants

One hundred and seventy-five healthy adults were scanned including 63 healthy, young participants (24 men and 39 women mean (±SD) age = 25.79 (2.70); mean (±SD) years of education = 15.46 (1.97); all right handed), and 112 healthy, old participants (4530 men and 59 women; mean (±SD) age = 65.47 (2.89); mean (±SD) years of education = 16.04 (2.64); all right handed). Participants were recruited using market-mailing procedures to equalize the recruitment process for young and old adults. Participants who responded to the mailing were telephone screened to ensure they met basic inclusion criteria (right handed, English speaking, no psychiatric or neurological disorders, normal or corrected-to-normal vision). All participants found eligible via the initial telephone screen were further screened in person with structured medical, neurological, psychiatric, and neuropsychological evaluations to ensure that they had no neurological or psychiatric disease or cognitive impairment. The screening procedure included a detailed interview that excluded individuals with a self-reported history of major or unstable medical illness, significant neurological history (e.g., epilepsy, brain tumor, and stroke), history of head trauma with loss of consciousness for greater than 5 min or history of Axis I psychiatric disorder (American Psychiatric Association, 1994). Individuals taking psychotropic medications or medications that influenced cognition were also excluded. Global cognitive functioning was assessed with the Mattis Dementia Rating Scale, on which a score of at least 133 was required for retention in the study (Mattis, 1988). Informed consent, as approved by the Internal Review Board of the College of Physicians and Surgeons of Columbia University, was obtained in writing prior to study participation, and after the nature and risks of the study were explained. Participants were compensated for their participation in the study.

### Behavioral Task

**FIGURE 1:**
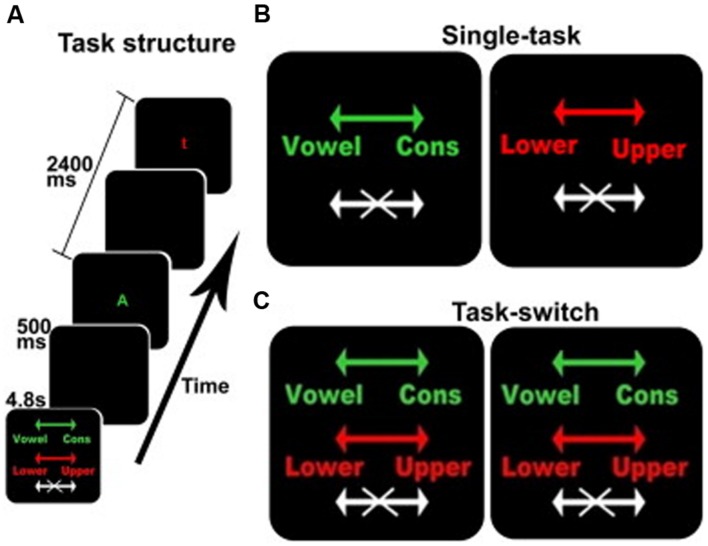
**The switching cognitive task used during fMRI scanning.** **(A)** An example of the beginning of a block including the instruction screen for a task-switching block, intertrial interval, a stimulus for the vowel/consonant task, intertrial interval, and a stimulus for the upper-/lower-case task. **(B,C)** The instruction screens for the single-task and the task-switching conditions, respectively. The colors served as task-cues: green for the vowel/consonant task, red for the upper-/lower-case task, and white for no-go trials. Arrows show the response-hand assignments: left for vowel/right for consonant and left for lower-case/right for upper case.

The behavioral task was derived from Experiment 2 in the task developed by Koechlin et al. (2003). This is an intrinsically cued task-switching paradigm with a no-go component where the color of each stimulus served as the task cue, see **Figure 1**. Participants were presented with a series of four conditions comprised of two single-task conditions and two identical task-switching conditions, with the duplication serving to match the number of trials for each discrimination between the single and switch-task conditions (see below). Each block was preceded by a 4.8 s instruction cue to inform the participant of the appropriate action for each stimulus. Each 33.6 s block, comprised 12 sequential letters (or trials) each presented for 1900 ms with an inter-trial time of 500 ms. Each stimulus was terminated when a response was made or when the trial deadline was reached. These trial dynamics were selected based on performance characteristics of the older adults in behavioral pilot studies, and deviated from Koechlin’s briefer presentations (Koechlin et al., 2003). Participants responded to each letter with a right-hand/left-hand button press or by making no action at all.

In addition to the four active conditions, there were two 33.6 s resting conditions when no stimuli were presented and no response was required. The two resting conditions were identical, but were separately enumerated to simplify description of the Latin Square design. Each resting block presented an instruction cue (“REST”) followed by a blank screen. During fMRI acquisition, each participant was given six repetitions of each of the four active and two resting conditions, for a total of 36 blocks. Conditions were presented in a 6 × 6 fully balanced Latin Square design. The fMRI data acquisition protocol requires stopping the scanner after every six blocks, typically requiring less than 30 s, resulting in the total session duration of approximately 26 min and a total of six fMRI runs with six blocks in each run.

In order to promote the scanning of participants in a stable behavioral and cognitive state, participants were pre-trained on the task and then tested on the entire paradigm in a quiet office prior to the MRI scanning session. Training consisted of giving between one and three blocks of each condition, with unlimited time to inspect the full instructions and the instruction cues preceding each block, and with auditory feedback indicating incorrect responses. Then participants were tested on the entire 6 × 6 Latin Square identical to the testing protocol described above (pre-scan phase).

### Stimulus Presentation

Task stimuli were back-projected onto a screen located at the foot of the MRI bed using an LCD projector. Participants viewed the screen via a mirror system located in the head coil and, if needed, had vision corrected to normal using MR compatible glasses (manufactured by SafeVision, LLC. Webster Groves, MO, USA). Responses were made on a LUMItouch response system (Photon Control Company) using the index fingers. Task administration and collection of RT and accuracy data were controlled using PsyScope 5X B53 (Macwhinney et al., 1997) running on a Macintosh G3/G4 iBook. Task onset was electronically synchronized with the MRI acquisition computer. A MellonIOLabs Systems USB Button Box provided digital input–output for the response system and synchronization with the MRI acquisition computer, as well as millisecond accurate timing of responses.

### MRI Data Acquisition

MRI images were acquired in a 3.0 T Philips Achieva Magnet using a standard quadrature head coil. A T1-weighted scout image was acquired to determine participant position. One hundred and sixty-five contiguous 1 mm coronal T1-weighted images of the whole brain were acquired for each participant with an MPRAGE sequence using the following parameters: TR 6.5 ms, TE 3 ms; flip angle 8°, acquisition matrix 256 × 256 and 240 mm field of view. Six functional scan sets were acquired, each of which included collection of 111 functional images acquired using a field echo echo-planar imaging (FE–EPI) sequence TE/TR = 20 ms/2000 ms; flip angle = 72°; 112 × 112 matrix; in-plane voxel size = 2.0 mm × 2.0 mm; slice thickness = 3.0 mm (no gap); 41 transverse slices per volume. Before the initiation of the executive task, four volumes were acquired and discarded to allow transverse magnetization immediately after radiofrequency excitation to approach its steady-state value. A neuroradiologist reviewed all T1 scans for potentially clinically significant findings, such as abnormal neural structure; no clinically significant findings were identified or removed.

### Image Pre-processing

All image pre-processing and statistical analyses used SPM8 (Wellcome Department of Cognitive Neurology). For each participant’s EPI dataset: images were temporally shifted to correct for slice acquisition order using the first slice acquired in the TR as the reference. All EPI images were corrected for motion by realigning to the first volume of the first session. The T1-weighted (structural) image was coregistered to the first EPI volume using mutual information. This co-registered high-resolution image was used to determine the transformation into a standard space defined by the Montreal Neurologic Institute (MNI) template brain supplied with SPM8. This transformation was applied to the EPI data and re-sliced using sinc-interpolation to 2 mm × 2 mm × 2 mm. Finally, all images were spatially smoothed with an 8 mm FWHM kernel (Mikl et al., 2008).

### Time-Series Analysis

First-level time-series analyses used a block-based model composed of epochs separately representing the single and switch-task conditions. Each epoch was convolved with a canonical model of the hemodynamic response function supplied with SPM8. Contrasts of the switch-task versus single task conditions were entered into the group-level mediation analysis.

### Gray Matter Volume

The T1-weighted anatomical images were first segmented into three tissue types: gray matter, white matter, and cerebrospinal fluid, using the unified segmentation routines in SPM8 (Wellcome Department of Cognitive Neurology; Ashburner and Friston, 2000, 2005; Good et al., 2001b). This procedure uses a single generative model to correct for image intensity non-uniformity (bias), registration with tissue class priors, and tissue classification. The result is a classification for each voxel based on the probability that it belongs to each tissue type. Each image segment therefore contains measures of tissue densities in each voxel location. The images were spatially normalized to the study specific normalization template using 12 degrees of freedom affine transforms and non-linear warping. Once warped, the images were modulated using the Jacobian determinant, which converts the density images into measures of absolute volume at each voxel location (Good et al., 2001b). The resultant modulated, spatially normalized gray matter probability maps were spatially smoothed with an isometric Gaussian smoothing kernel of 8 mm^3^ at its full-width at half-maximum (FWHM) to result in gray matter volume maps.

### Covariates

As a measure of whole brain gray matter volume, the proportion of intra-cranial volume occupied by gray and white matter was calculated. Total volume of gray matter plus white matter was divided by the sum of gray, white, and CSF volume to derive the intra-cranial brain fraction (Chard et al., 2002; Fotenos et al., 2005). This measure is termed normalized whole brain volume (nWBV) and was used as a covariate in the analyses. Sex differences are well established in global brain size differences, with males having larger total brain volumes (Good et al., 2001a); therefore, sex was also included in all analyses as a covariate.

### Mediation Analyses

The mediation model tested whether age related differences in global switch costs (total effects) could be partially explained by voxel-wise measures of gray matter volume and/or task related brain activation (indirect effects). This approach uses switch costs as an output measure that is assumed to be affected by brain function, brain structure, and other age-related effects. Testing for mediation involves fitting multiple linear regression models at every voxel of the brain and assessment of statistical significance using non-parametric statistics specifically designed for use with brain imaging data to correct for multiple comparisons

**FIGURE 2:**
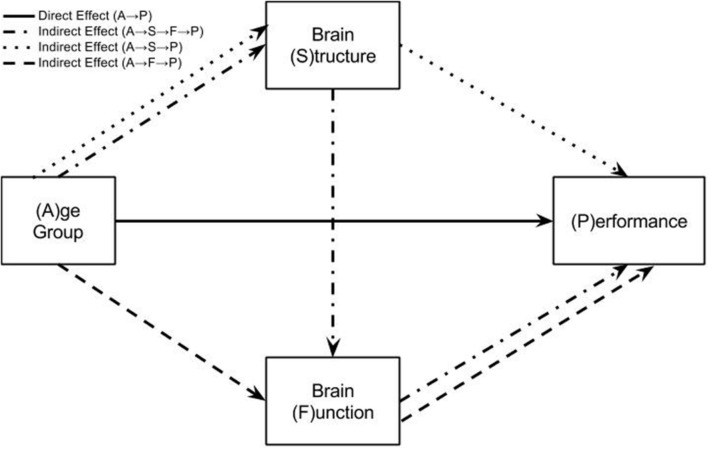
**Mediation model testing how age group differences in performance are explained by direct or indirect effects.** This model represents three regression equations fit at every voxel in the brain data. The solid arrow represents the direct effect of age group on task performance. This is the effect of age group on task performance after accounting for brain structure and function in the regression model. The three broken arrows represent the three possible indirect effects from age group to task performance. The dotted line is the pathway: Age Group → Brain Structure → Performance. The dashed line is the pathway: Age Group → Brain Function → Performance. The dot-dash line is the pathway: Age Group → Brain Structure → Brain Function → Performance. The sum of the direct and three indirect effects of age group on task performance represent the total effect of age group. This total effect may also be calculated by simply regressing age group onto task performance.

The mediation model used in this study includes two mediators of the relationship between age and task performance. These are structure (S) measured as gray matter volume and functional brain activity (F). The indirect effect of age on switch costs may therefore arise through four pathways (see **Figure 2**):

*A* → (*P*|*S*,*F*), the direct effect of age on performance after accounting for structure and function.

*A* →*S* → (*P*|*F*), the indirect effect of age on performance as mediated by structure after accounting for the effects of brain function.

*A* →*F* → (*P*|*S*), the indirect effect of age on performance as mediated by brain function after accounting for the effects of structure.

*A* →*S* →*F* →*P*, the indirect effect of age on performance as mediated by structure and brain function.

The regression equations for testing this model are:

1 P = c ⋅ A + ε_1_
2 S = a ⋅ A + ε_2_
3 F = b ⋅ S + d ⋅ A + ε_3_
4 P = e ⋅ F+f ⋅ S + c′⋅ A + ε_4_

The total effect of age on performance is *c* in Eq. 1. This is the effect that the mediation model is trying to explain with measures of gray matter volume and brain activity. This total effect of age on switch costs is therefore split into the direct effect and three indirect effects. The indirect effects are calculated by multiplying regression parameters together.

• Total effect: *c*
• Direct effect: *c*′
• Indirect effect through *S*: *a* ⋅*f*
• Indirect effect through *F*: *d* ⋅*e*
• Indirect effect through *S* and *F*: *a* ⋅*b* ⋅*e*
• Total effect = direct effect + all indirect effects:

$$c=c^{\prime }+a\cdot b\cdot c+a\cdot f+d\cdot e$$

After fitting Eqs 2–4 at each voxel in the brain, the indirect effects were calculated. Testing voxel-wise significance of indirect effects used permutation inference and the ‘single threshold’ permutation test (Nichols and Holmes, 2002; Winkler et al., 2014) using threshold free cluster enhancement (TFCE; Smith and Nichols, 2009). This approach accounts for multiple comparisons. For this method, 2000 permutations were performed where group assignment was randomly shuffled for each permutation. All analyses used the publically available and modifiable “Process Models for Neuroimaging” toolbox^1^ developed by the author JS. This toolbox implements the methods of Preacher and Hayes for use with neuroimaging data. An additional threshold was used based on the percentage of total effect of age on switch costs that was accounted for by the indirect effects. The effects had to be at least five percent of the total effect. This threshold limits results to brain regions having significant effects that are also relatively large.

## Results

### Behavioral Results

Global switch costs for correct trials were greater for the old adults than the young when tested with linear regression of age group onto switch costs: β = 0.0799, *t*(173) = 3.90, *p* < 0.001, mean (SD) of the young: 0.22 (0.083) seconds and for the old: 0.30 (0.15) seconds. Levene’s test for equality of variance demonstrated that the variance was significantly higher for the old adults, *F*(1,173) = 19.79, *p* < 0.001. After accounting for covariates of sex and nWBV this effect was still significant (β = 0.067, *t*(171) = 2.21, *p* < 0.05). The β-value here is the total effect *c* from Eq. 1. Analyses only focused on global switch costs since they best matched the block-design used for analysis of the fMRI data.

### Brain Imaging Results

All analyses were performed in brain regions demonstrating increased task-related signal change in at least one of the groups using uncorrected two-tailed *t*-tests of α < 0.05 intersected with a gray matter probability mask of 0.5. Inclusion of this mask ensures that results are limited to brain locations where there is a significantly large effect size for the brain activation measure in regions comprised largely of gray matter. This helps minimize spurious findings and provides an additional control over type I error.

**FIGURE 3:**
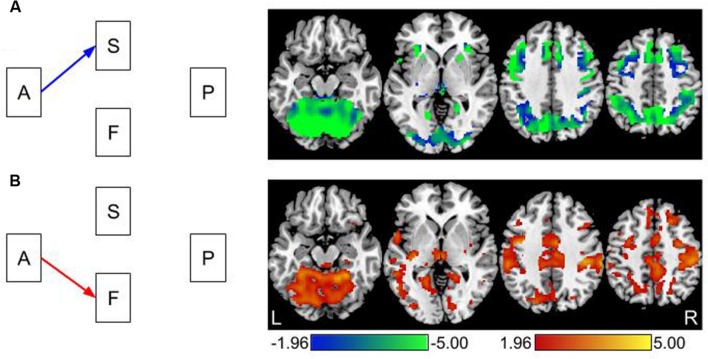
**Direct effects of age on gray matter volume and brain activity.** **(A)** The effect of age on gray matter volume using voxel based morphometry (VBM). All effects are in the negative direction; older adults have lower gray matter volume than young adults. This is represented with a blue arrow in the path model on the left. **(B)** The effect of age on brain activation. All effects are in the positive direction; older adults have greater levels of task-related brain activation than young adults. This is represented with a red arrow in the path model on the left. Images are thresholded using a two-tailed test of *p* < 0.05, *Z* > 1.96.

**FIGURE 4:**
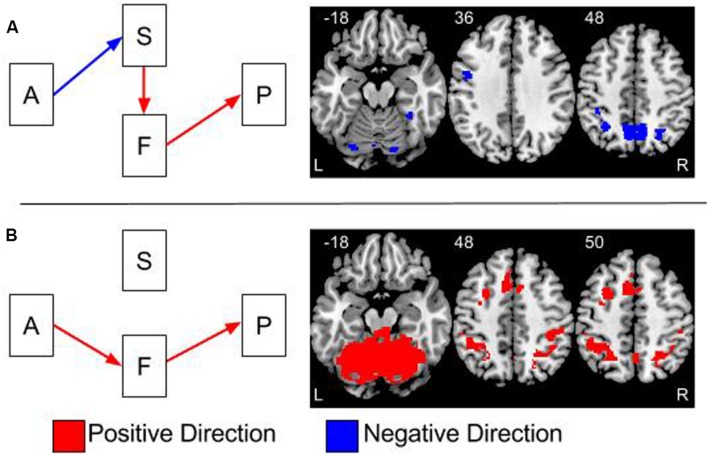
**Overlays of the two significant indirect effects in the model.** **(A)** The indirect effect between age group and performance, via gray matter volume and brain function. **(B)** The indirect effect between age and performance via brain function. The effects in **(A,B)** are thresholded at a two-tailed alpha <0.05 corrected for multiple comparisons using 2000 permutations and threshold free cluster enhancement (tfce). The colors of the arrows on the path models represent the direction of the effects. Blue arrows represent negative effects and red arrows are positive effects. The colors of clusters of activations in the brain overlays represent the direction of the indirect effects. These indirect effects are calculated by multiplying the effects at each step in the pathway together. Therefore, the one negative and two positive effects in **(A)** result in an overall negative effect.

Effects of age group on gray matter volume and brain activation are shown in **Figure 3**. These results represent the first step in the mediation analysis on the left hand side of **Figure 2**. They also demonstrate the magnitude and direction of the effects in the mediation analyses shown in **Figure 4**. The liberal threshold used here is to demonstrate that age group effects occur in the data. This threshold has no impact on the indirect effects reported later, which use strict correction for multiple comparisons.

Advancing age had a negative effect on gray matter volume throughout the brain. Therefore, as age increased, gray matter volume decreased, see **Figure 3A**. Advancing age was related to increased levels of task related signal change. In other words, older adults had higher levels of brain activity than younger adults, see **Figure 3B**.

### Mediation Results

Mediation analyses tested the three indirect pathways between age group and global switch costs. Results were thresholded at two-tailed α < 0.05 correcting for multiple comparisons using the TFCE method with the ‘single threshold’ permutation test with 2,000 permutations. The TFCE method eliminates the need for cluster threshold allowing results to be interpreted with a single threshold instead of a combination of height and cluster size thresholds. Results from the indirect effects are shown in **Figure 4** and in **Tables 1** and **2**. The tables include the proportional size of the indirect effects and the *t*-values for the within and between age group differences in brain activation.

**Table 1:** Age → Gray Matter Volume → Brain Activity → Switch costs.

Region	H	BA	*x*	*y*	*z*	*k*	Y (*t*)	O (*t*)	O > Y (*t*)	%IND
Angular	L	40	-32	-50	38	166	4.22	6.52	0.40	-5.12
Sup. Parietal	L	7	-20	-62	42	-	1.97	6.60	2.03	-5.27
Inf. Parietal	L	7	-28	-60	42	-	4.43	7.25	0.78	-5.34
Angular	R	7	28	-62	46	105	2.16	5.37	1.33	-12.17
Angular	R	40	36	-54	40	-	3.17	4.79	0.72	-5.02
Precuneus	L	7	-4	-66	42	453	1.94	6.76	1.99	-6.02
Precuneus	R	-	4	-58	44	-	0.67	4.34	1.77	-5.64
Precuneus	L	-	-6	-58	44	-	1.34	5.58	2.17	-6.70
Inf. Parietal	L	40	-38	-38	46	22	0.62	3.36	1.32	-5.52
Cerebellum	L	18	-20	-78	-22	51	0.82	3.73	1.64	-6.10
Cerebellum	L	19	-28	-76	-20	-	0.93	4.60	2.25	-6.47
Cerebellum	R	18	18	-78	-18	43	1.21	4.40	1.90	-7.54
Inf. Occipital	L	19	-30	-84	-6	16	2.32	5.44	1.81	-5.52
Inf. Occipital	R	19	32	-88	-4	22	4.13	5.84	0.81	-6.52
Vermis	R	-	4	-64	-12	41	-0.32	2.95	2.07	-9.04
Fusiform	R	37	34	-40	-20	24	2.89	2.33	-0.37	-7.36
Fusiform	L	19	-38	-66	-16	10	1.56	3.84	1.70	-5.26
Fusiform	L	37	-34	-58	-16	-	0.75	5.10	2.91	-5.23
Precentral	L	-	-46	-4	38	47	-1.90	3.20	3.43	-11.07
Cerebellum	R	-	20	-56	-28	17	0.54	3.18	1.44	-6.35
Lingual	L	17	-6	-68	6	11	-0.24	2.26	1.46	-6.14
Cerebellum	L	-	-16	-60	-28	10	0.32	4.74	2.54	-6.64


*Y (*t*), O (*t*), and O > Y (*t*) are t-values from within and between group tests for the size of the brain activation effect. %IND is the percentage, with direction, of the total effect of age on performance that this indirect effect accounts for. BA, Brodmann Areas; – refers to local maxima or locations without atlas labels, Inf, inferior; Sup, superior.*

**Table 2:** Age → Brain Activity → Switch costs.

Region	H	BA	*x*	*y*	*z*	*k*	Y (*t*)	O (*t*)	O > Y (*t*)	%IND
Cerebellum	R	-	12	-40	-32	8585	-0.49	3.18	2.33	11.99
Cerebellum	R	-	4	-36	-32	-	0.45	1.97	1.12	11.41
Cerebellum	L	-	-10	-36	-32	-	0.22	2.22	1.28	12.83
Mid. Occipital	L	19	-26	-86	18	529	-0.94	2.81	2.45	10.07
Mid. Occipital	L	19	-34	-84	20	-	-1.88	2.34	2.87	8.94
Mid. Occipital	L	19	-24	-80	24	-	-1.54	2.06	2.39	10.41
Precentral	L	6	-46	0	28	125	0.84	3.77	1.55	20.81
Precentral	L	6	-42	-4	34	-	-0.22	4.64	2.94	22.87
Precentral	L	6	-52	0	34	-	-0.63	2.41	1.97	26.27
Supramarginal	L	40	-36	-40	36	506	1.17	4.92	1.86	8.89
Inf. Parietal	L	40	-44	-36	36	-	0.67	3.37	1.34	8.38
Sup. Parietal	L	40	-30	-46	38	-	3.08	6.12	0.97	9.23
Supramarginal	R	40	34	-40	38	218	0.11	3.33	1.96	9.09
Supramarginal	R	40	40	-34	38	-	-1.34	2.88	2.81	11.01
Supramarginal	R	2	50	-28	40	-	-2.42	2.28	3.44	10.26
Mid. Cingulum	L	32	-6	22	40	240	3.24	6.09	1.67	23.46
Sup. Med. Frontal	L	8	-2	30	42	-	1.16	4.14	1.96	27.15
Mid. Cingulum	L	32	-6	12	44	-	1.19	4.10	2.06	25.23
Precuneus	R	7	2	-60	48	69	1.57	5.53	1.79	18.26
Precuneus	R	-	10	-56	52	-	0.96	5.26	2.52	12.79
Precuneus	L	7	-2	-56	54		-0.03	5.38	2.65	13.28
Mid. Frontal	L	6	-26	2	48	89	2.23	5.55	2.19	19.63
Mid. Frontal	L	6	-28	10	48	-	3.05	4.35	1.26	21.23
Mid. Frontal	L	-	-20	6	52	-	1.83	4.81	1.98	21.36


*Y (*t*), O (*t*), and O > Y (*t*) are t-values from within and between group tests for the size of the brain activation effect. %IND is the percentage, with direction, of the total effect of age on performance that this direct effect accounts for. The total effect was 0.067 allowing calculation of the indirect effect size. BA, Brodmann Areas; – refers to local maxima or locations without atlas labels, Inf, inferior; Med, medial; Mid, middle; Sup, superior.*

Mediation results were significant in two out of the three possible pathways, age to structure to function to switch costs and age to function to switch costs, see **Figure 4**. Both pathways partially explained the negative effect of age group on switch costs.

#### Age to Structure to Function to Switch Costs Pathway

This effect is shown in **Figure 4A** and **Table 1**. All indirect effects along this pathway were **negative**. Therefore, as age increased, gray matter volume decreased; gray matter volume and brain activation were positively related and brain activation and switch costs were also positively related. The bilateral precuneus had a significant effect and brain activity in these regions were significantly greater than zero for the old adults; however, not for the young adults. Results within the bilateral parietal cortex had highly significant brain activity for the old adults and lower, but also significantly greater than zero, brain activity for the young. Regions of the left precentral gyrus, cerebellum, fusiform, and occipital cortices also had significant indirect effects.

#### Age to Function to Switch Costs Pathway

This effect is shown in **Figure 4B** and **Table 2**. Results from testing this pathway were exclusively masked with the results from the age to structure to function to switch costs pathway to highlight results specific to this pathway only. Brain regions demonstrating a significant indirect effect along this pathway were all in the **positive** direction. Therefore, as age increased, brain activation increased and brain activation and switch costs were positively related. Large regions within the cerebellum and occipital cortex demonstrated this effect. Additional regions included the left precentral gyrus, bilateral supramarginal, bilateral parietal, precuneus, middle cingulate extending to medial superior frontal gyri and the left middle frontal gyri. Significant effects along this pathway are controlled for gray matter volume.

## Discussion

A full brain exploration identified whether the effects of age group on cognitive control, as measured with global switch costs, are related to regional gray matter volume and brain activation during the task. Results show that advanced age has a universal negative effect on gray matter volume throughout the brain. Furthermore, brain activity was always greater in the old adults compared to the young. The total effect of age on switch costs was positive: increased age was related to increased switch costs. The analyses parsed this total effect into three indirect effects through the mediating pathways of gray matter volume and brain activity, and a direct effect. The direct effect is the leftover relationship between age and switch costs after accounting for both neural variables. Therefore, the sum of the direct and indirect effects equals the total effect.

The results of this study converge on many findings from the literature relating age, neural measures, and switch costs. The current results reflect the presence of multiple age-related neural effects having relationships with task performance. At the group level, performance on the cognitive control task declined with advancing age. In all regions where the indirect effect via structure and function were significant, **Figure 4A**, they were in the same direction. Age increased, gray matter volume decreased, brain activation increased and switch costs worsened. It is possible that the increased levels of brain activity were in response to the age-related declines in gray matter volume. While a number of previous studies reported lower gray matter volume (Raz and Rodrigue, 2006; Fjell and Walhovd, 2010) and increased brain activation with aging (D’Esposito et al., 2003; Lustig et al., 2009), the current work is novel by demonstrating direct and indirect associations among age, gray matter volume, brain activation and switch costs within a single analysis.

The effect of advancing age on switch costs is positive; however, the negative indirect effect implies that as age increases, switch costs decrease. Within the statistical literature this represents inconsistent mediation or statistical suppression (MacKinnon et al., 2000). Therefore, controlling for brain function increases the negative effect of age-related declines in gray matter on switch-costs. In other words, if there were no brain function in any of these regions gray matter declines would have a larger effect on switch costs than they actually do. Therefore, the presence of increased brain function lessens, or suppresses, the negative effects of gray matter on task performance.

However, explaining these findings as statistical suppression does not eliminate the fact that gray matter volume and brain function are positively related. Likewise, brain function and switch costs are positively related. The directionality of these effects fits well with current theories of cognitive aging. Declining gray matter volume is thought to decrease neural efficiency and capacity (Stern, 2009; Steffener and Stern, 2012; Barulli and Stern, 2013). Decreased neural efficiency and capacity are reflected in the current data as age-related increases in switch costs due to decreased efficiency. Likewise, neural capacity, the maximal level of brain function, declines as gray matter volume declines.

In regions where the indirect effects via function alone were significant, **Figure 4B**, the results were in the positive direction. Age-related increases in switch costs were related to age-related increases in brain activity within the cerebellum. This suggests that the increased brain activity in the cerebellum is related to switch costs but independent of the effects of gray matter volume. The cerebellum was identified in previous work with this task using multivariate analyses (Gazes et al., 2012). In patients, who underwent surgical damage to the cerebellum for tumor removal, there were increased switch costs, despite normal learning of the task (Berger et al., 2005).

The cingulate and supplementary motor areas (SMA) were also highlighted by the age to function to switch costs pathway. These regions were nodes in a previous multivariate analysis of brain activation using the same task and expression of this pattern was related to switch costs (Gazes et al., 2012). Increased activation in older adults was also related to increased response time in a similar task-switching experiment (Hakun et al., 2014). In a motor switching task, increased activation in the SMA was interpreted as evidence for an alternative strategy being used by the older adults for task completion (Coxon et al., 2010). Within the current results, the young adults had non-significant levels of brain activation in these regions, while activation levels were significant for the old age group, see the Y(*t*), O(*t*), and O > Y (*t*) columns of **Tables 1** and **2**. This supports the idea of Coxon et al. (2010) that the medial PFC regions are used as an alternative strategy. Structural measures in this region have also been linked to cognitive performance. The gray matter volume and white matter concentration within the anterior cingulate are shown to be related to one’s ability to control one’s own brain activity in a study of biofeedback in young adults (Enriquez-Geppert et al., 2013). The volume of the anterior cingulate is also related to executive task performance in older adults (Elderkin-Thompson et al., 2009). These results suggest that the medial PFC is an important brain region for dual task processing and the effects of age on the structure, function and structure-function interactions are complicated and require more in-depth study.

The overall results of this work demonstrate that some of the age-related effects on switch costs can be attributed to individual differences in gray matter volume and brain activation. While this finding is not surprising, the current results demonstrate that not all age effects on neural measures translate into task performance effects. Age group differences in gray matter volume and brain activation were broadly distributed to large areas of the brain as shown in **Figure 3**. The mediation results demonstrate that the age-related neural effects were only related to task performance in a relatively small subset of brain regions. Areas of the parietal cortex, precuneus, cerebellum, occipital and precentral gyrus represent locations where the combined age-related structural and functional effects appear to have some impact on switch costs.

Interestingly, the results of this study also demonstrate a subset of brain regions where the effect of age group on switch costs was mediated by brain activation after controlling for gray matter volume, **Figure 4B** and **Table 2**. This suggests that the age effect on brain activation in these regions is the result of other unmeasured processes. Interestingly, the brain regions highlighted from this pathway include multiple prefrontal cortical (PFC) regions. The pathway including gray matter volume, and controlling for brain activation, was not significant for any brain regions.

The developers of the executive context factor task used in this study posited a cascade of executive processing through the prefrontal cortex (Koechlin et al., 2003). The implicated brain regions included premotor, caudal, and rostral lateral PFC regions. The age group effects in brain activation from the current work demonstrate amplitude differences within these brain regions, **Figure 3**. Except for the precentral gyrus, these regions do not show up in the age to structure to function to switch costs mediation analysis. However, some of these regions do show up in the age to function to switch costs mediation pathway. This demonstrates that age related differences in the brain activation in these regions are related to switch costs independent of the effect of age on gray matter. This suggests the presence of a different mechanism that is causing these effects. It is plausible that these brain regions are compensating for effect of age-related declines in gray matter volume elsewhere in the brain.

To test the hypothesis that brain activation is compensatory for gray matter volume effects elsewhere in the brain, functional connectivity analyses could be integrated into mediational analyses. This interpretation of greater brain activation being compensatory is tenuous due to the declining task performance as a function of the increased brain activation. This supports the interpretation that not all additional brain activation is good for performance (see Steffener and Stern, 2012, for a review). Furthermore, usage of extra brain resources could represent a larger network of functioning nodes that increase network transit time thereby increasing response times. These interpretations reflect the importance of integrating multiple physiological measures of the aging process to understand cognitive aging (Grady, 2012). The use of mediation analysis demonstrates one approach to better understanding the neural mechanisms of cognitive aging.

The old age group had larger variability in their task performance. Their standard deviation was significantly different from the young, as per Levenes’ test of equal variance. It is possible that the larger variance in the old group represents cumulative effects of individual differences in lifetime exposures. Individual differences could alter brain measures or performance (Grady, 2012) and they may affect the interrelationships between the variables (Steffener and Stern, 2012). Future directions will begin exploring the roles of individual differences in lifetime exposures to determine if they explain some of the increased variance in the older adults. A first step, will parallel our previous work by including measures of education and verbal abilities (Steffener et al., 2014). Future work will carefully and completely investigate the role of education along with genetics, leisure activities, physical activity, and nutrition.

A major concern for mediation analyses is the assumed directionality of causal inference. It was assumed that task performance is the outcome measure that is indirectly or directly affected by the neural measures. The opposite approach is also possible (Zhu et al., 2014). Although potentially contentious, we assumed that measured brain activation was influenced by measured gray matter volume. It is also plausible that long-term differences in brain activation may result in structural reorganization. Another concern in making causal inferences was outlined in Maxwell et al. (2011) and led to a debate within the statistical literature (Imai et al., 2011; Shrout, 2011; Thoemmes, 2015). The authors demonstrated cross-sectional analysis might give biased estimates of indirect effects. They further discuss that the most appropriate tests of caudal indirect effects are longitudinal models with time delays in the relationships among independent, mediating and dependent variables.

In the current work, the true longitudinal effects are unknown. We currently do not know how fMRI measures of brain activity adapt to age-related changes in gray matter volume. We also do not know whether age-related changes in brain activity would affect gray matter volume. It is also reasonable to assume that task performance is the result of neural operations occurring during the performance on that task. The current work also used tight control of multiple comparisons such that no indirect effect would be found if age was merely independently affecting the neural and performance measures. Thus, an indirect effect, while possibly biased with regard to longitudinal models, is unlikely to have arisen by chance. Interpretation of the current results as causal requires follow-up longitudinal data collection; however, the identified indirect effects between age and switch costs are valid despite the use of cross-sectional data.

These concerns are present in all mediation analyses. However, the use of mediation analyses with neuroimaging data has been increasing in recent years. Some of the first work with voxel-wise mediation comes from Wager et al. (2008) and his multilevel mediation analyses. Other recent work exploring mediating effects within aging research investigated hippocampal volume (Rodrigue et al., 2013) and white matter tract integrity (Gold et al., 2008; Voineskos et al., 2012). This trend of using imaging data to explain age-related differences in cognition and task performance will have a great impact on the field. It provides greater insight into understanding the heterogeneity of cognitive aging and develop the understanding of the neural mechanism underlying healthy aging and the lifestyle and behavioral choices people make leading them to maintained cognition in late life.

Mediational analyses have benefits over regression and correlational studies, because they allow plausible causal directions and integrate multiple measures into a single model. Due to low computational burden, voxel-wise measures are typically used as independent variables in simple regression models. The current mediational model uses the brain measures as dependent variables predicting cognitive outputs. The high computational burden of this approach is greatly alleviated with modern computing facilities. Mediation analyses also allow straightforward and understandable testing of theories. Current theories of cognitive aging call for the integration of multiple physiological measures into a single model with hypothetical causal relationships. Mediation models explicitly test such models and allow the inclusion of moderating effects.

## Conclusion

These overall results demonstrate that age-related effects on switch costs can be attributed to individual differences in gray matter volume and brain activation. The regional results demonstrate that some, but not all, of the age-related differences in brain activation are related to gray matter volume differences. Age-related differences in brain activation, independent of gray matter volume, may represent compensation. These additional functional resources may be employed to compensate for age-related gray matter volume declines affecting the function elsewhere in the brain. Future inter-regional explorations of structure–function–performance relationships are required to address these questions.

## Author Contributions

JS: conceptualized analyses and performed analyses, interpreted results, wrote manuscript. YG: interpreted results, wrote manuscript. CH: conceptualized analyses. YS: interpreted results.

## Conflict of Interest Statement

The authors declare that the research was conducted in the absence of any commercial or financial relationships that could be construed as a potential conflict of interest.

## Footnotes

1 https://github.com/steffejr/ProcessModelsNeuroImage
